# Accuracy of TomoEDGE dynamic jaw field widths

**DOI:** 10.1002/acm2.12418

**Published:** 2018-07-29

**Authors:** Patrizia Urso, Nathan A. Corradini, Cristina Vite

**Affiliations:** ^1^ Radiotherapy Center Clinica Luganese Moncucco SA Lugano Switzerland

**Keywords:** dynamic jaws, field width, TomoEDGE™, tomotherapy

## Abstract

Dynamic jaw delivery on the TomoTherapy H‐series platform, entitled TomoEDGE™, is an effective tool to decrease the patient dose along the superior and inferior edges of the treatment target. The aperture of the TomoTherapy jaws, that is, field width (FW), defines the longitudinal dose profile. A consistent FW dose profile is an important quantity for accurate and reproducible dose delivery in TomoTherapy. To date, no evaluation has been made of the accuracy and precision of the dose profiles produced by dynamic jaws. This study aims to provide a long‐term evaluation of the dynamic jaw FW dose profiles obtained on TomoTherapy utilizing the TomoTherapy Quality Assurance procedure (TQA). A total of 840 dose profiles were measured during 84 TQA procedures, performed over a 2‐yr period. The full width at half maximum (FWHM) and constancy of the FW dose profile measurements were analyzed and compared with the tolerances proposed by AAPM Task Group 148 (TG‐148) and those used by the manufacturer. The FWHM evaluation showed that the FWs > 2.0 cm respect the TG‐148 tolerance of 1%, while the asymmetric FWs ≤ 2.0 cm were outside the limit in 17.3% of measurements. Constancy results evaluated along the full profiles showed that 95.2% of measurements were within 3% of the baseline for symmetric FWs and 94.8% of measurements were within 4% of the baseline for asymmetric FWs. In conclusion, the analysis confirms the accuracy and precision of TomoEDGE™ technology in jaw positioning. This study has identified the potential to establish an appropriate QA tolerance for the asymmetric FWs used in dynamic jaw movement. Finally, the clinical significance of the observed discrepancies should be studied further to understand the dosimetric effect on patient treatments.

## 1 INTRODUCTION

**Figure 1:**
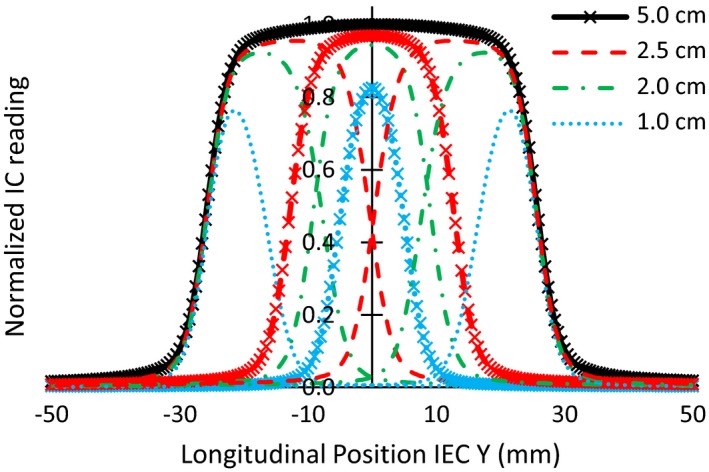
TomoEDGE™ commissioned longitudinal profiles. The TomoEDGE™ beam model utilizes 10 FWs for dose calculation. The original beam model uses 3 symmetric FWs, those denoted with “x” markers, whereas dynamic jaw motion additionally utilizes those shown with dotted and dashed lines.

The introduction of a sliding‐window dynamic jaw motion during treatment, entitled TomoEDGE™, has been a significant technological change to the TomoTherapy^®^ platform (Accuray Inc., Sunnyvale, CA).^1^ This advanced delivery modality was conceived with the aim of reducing the dose penumbra in the cranial‐caudal direction, allowing for more treatments with a 5.0 cm field width, and subsequently reducing treatment beam‐on times. In TomoEDGE™, the front and back secondary collimators, known as jaws, move individually in the *Y*‐axis direction (ie, longitudinal couch direction) as defined by the International Electrotechnical Commission (IEC) coordinate system (IEC Y). A load‐side encoder for each jaw provides independent absolute position and velocity feedback. Thus, a sliding‐window open‐and‐close jaw movement is performed at the superior and inferior aspects of the treatment target during irradiation. The aperture of the front and back jaws defines the longitudinal beam dose profile, which TomoTherapy also calls a field width (FW). Dynamic jaw motion requires the introduction of a new beam model with additional FWs for accurate dose calculation. In fact, the TomoEDGE™ beam model uses 10 longitudinal beam profiles, four symmetric and six asymmetric, while the original static jaw beam model required only three symmetric profiles (Fig. 1). Consistent FW profiles, symmetric and asymmetric, are necessary for accurate and reproducible dynamic dose delivery. It has been shown that the full width at half maximum (FWHM) of the FW profiles was an important variable directly linked to the delivered dose during TomoTherapy treatments.^2^ The AAPM task group on quality assurance of TomoTherapy notes the importance of measured FWHMs and recommends a careful monitoring of the parameter to ensure agreement with the beam model.^3^

To date, clinical advantages of TomoEDGE™ have been studied and reported, demonstrating a decrease in dose penumbra longitudinal to the target.^4^, ^5^ Furthermore, reduction in clinical treatment times with the use of TomoEDGE™ has been reported in multiple studies.^4^, ^6^ However, no studies have evaluated the TomoEDGE™ technology concerning jaw positioning accuracy and precision.

The aim of this work was to evaluate the performance of the dynamic jaws by measuring all 10 FWs commissioned on TomoEDGE™ on an approximate weekly basis for a 2‐yr period. A comparison of FWHM was used to estimate the accuracy and precision of jaw positioning, while beam profile constancy was measured to increase the evaluation sensitivity to changes in beam shape. Ultimately, these data provide a useful perspective for a critical assessment of both the tolerance values indicated by the AAPM TG‐148 report on quality assurance of TomoTherapy and those used by the manufacturer.

## 2 MATERIALS AND METHODS

**Figure 2:**
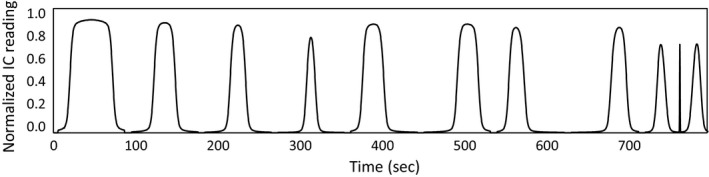
Example plot of a TQA Field Width Dynamic Jaws measurement. The nominal FWs are measured in the following order: 5.0, 2.5, 2.0, 1.0, 2.5 −IEC, 2.5 +IEC, 2.0 −IEC, 2.0 +IEC, 1.0 −IEC, 1.0 +IEC.

This study was performed on a TomoHDA machine and used the TQA Field Width Dynamic Jaws procedure, which sequentially automates open‐field measurement of all 10 longitudinal FW profiles (see example in Fig. 2). An Exradin A1sl ion chamber (IC) (Standard Imaging, Middleton, WI) was inserted into a solid water slab phantom at a depth of 1.5 cm while the phantom was placed at a source‐to‐surface distance of 85 cm. The couch height was adjusted within the bore to ensure alignment of the solid water slab with the green lasers and compensate for sag. The same ion chamber and solid water slabs were used throughout the study to reduce the intrinsic variability in measurements. The longitudinal profiles were acquired with a couch speed of 1 mm/s at a sample rate of 100 ms using a TomoElectrometer in conjunction with TEMS software (Accuray Inc., Madison, WI). The procedure was run 84 times, collecting 840 FW profiles, spanning a 2‐yr period from June 2013 to June 2015. Throughout the period of the study, machine QA related to mechanical alignment and beam parameters was performed rigorously in accordance with the frequency recommended in TG‐148.

Matlab software was used to analyze the measured FW dose profile data and to compare with those of the TomoEDGE™ beam model reference profiles, that is, the “gold standard” (GS), provided by the manufacturer. Measured data were normalized to their maximum values and the FWHM was calculated for each profile. Specifically, longitudinal positions of half‐maximum values were interpolated linearly between the 0.1 mm measurement steps to increase sensitivity of the FWHM estimate. The comparison of the FWHM results was interpreted using the 1% tolerance limit recommended in the AAPM TG‐148 report.

**Figure 3:**
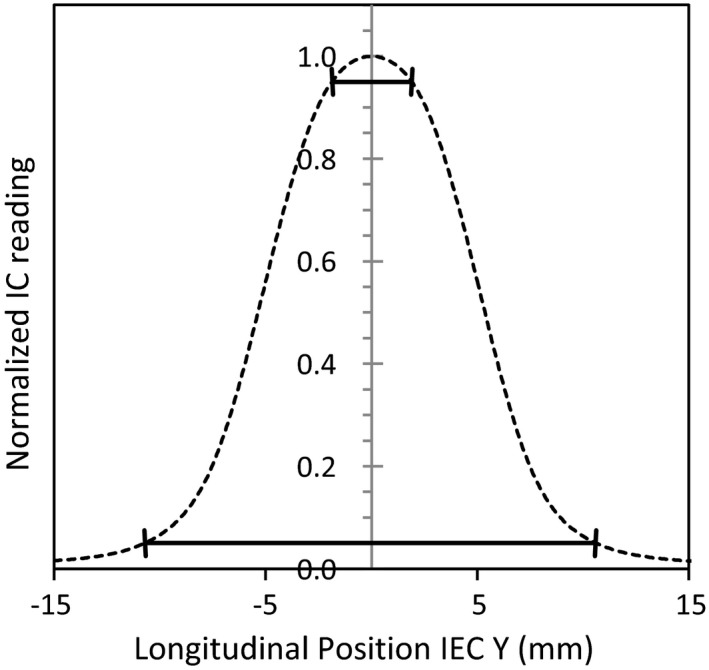
Example 1.0 cm FW profile that shows the chords between which the beam profile constancy was calculated. Peak constancy between the 95% values and full profile constancy between the 5% values.

In addition, measured FW datasets were resampled using the same y‐positions as GS reference data for beam profile constancy calculation. Beam profile constancy of the FWs was evaluated as described in Section II.A of TG‐142 report on quality assurance of medical accelerators^7^ using the GS profiles as the baseline for comparison, that is,

$$constancy=\frac{1}{N}\cdot \sum_{i=1}^{N}\left|\frac{MP_{i}-GSP_{i}}{GSP_{i}}\right|\cdot 100\%,$$

where MP_*i*_ and GSP_*i*_ are off‐axis ratios at measured and GS reference points, respectively, at profile point *i*,* N* is the number of profile points. Two constancy calculations were made: (a) between the 5% values for the full beam profile shape, and (b) between the 95% values in the peak region of the profile maximum. An example of the chords used to calculate beam profile constancy can be seen in Fig. 3.

## 3 RESULTS

**Figure 4:**
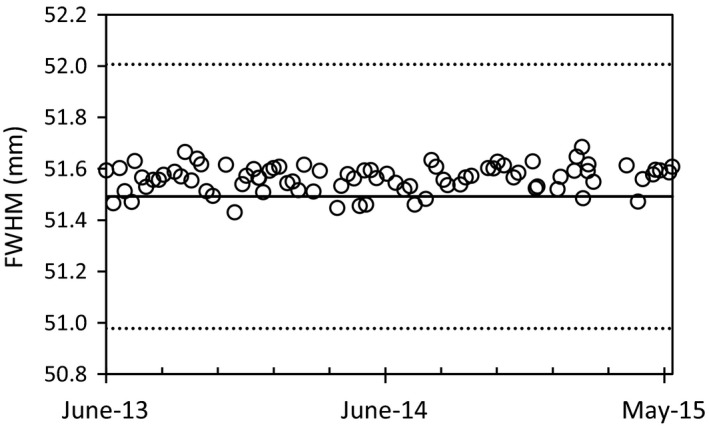
Timeline plot of the FWHM measurements for the 5.0 cm jaw setting. Dotted lines indicate the ±1% from GS reference. FWHM values are shown along the vertical axis in mm.

**Figure 5:**
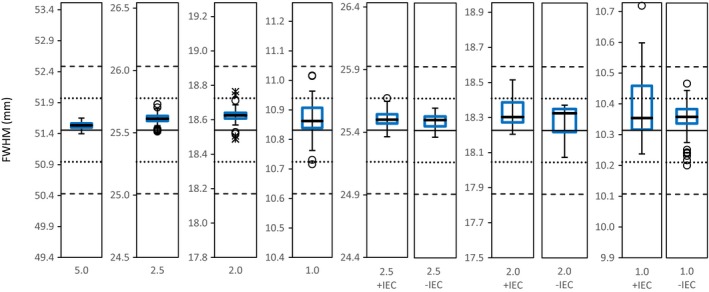
Tukey box plots of measured FWHMs for the nominal field width (FW) dose profiles in the TomoEDGE™ beam model. The box plots are illustrated using a window of ±4% of gold standard reference values (GS Ref.), which are indicated by the central solid lines. Dotted and dashed lines, respectively, indicate the ±1% and ±2% from GS Ref. FWHM values are along the vertical axis in mm.

An example of a FWHM timeline trend is shown in Fig. 4, where the 5.0 cm FW is reported. Results of the FWHM comparison are reported in Table 1. The mean reported FWHMs are observed to be within the TG‐148 1% tolerance recommendation. A systematic difference of the FWHMs from reference values can be seen throughout all the profiles. More specifically, the observed systematic difference in FWHM averaged 0.07 mm for all FWs, with a maximum absolute difference of 0.10 mm for the 2.0 +IEC FW and a maximum relative difference of 0.67% for the 1.0 +IEC FW. Profile standard deviations showed the smaller and asymmetric FWs to have a larger variation in absolute difference in measured FWHM. In reference to the AAPM TG‐148 1% recommendation, individual FWHM measurements were outside tolerance for 22.6% (*n* = 19), 11.9% (*n* = 10), 28.6% (*n* = 24), 7.1% (*n* = 5) of all cases for the 2.0 +IEC, 1.0, 1.0 +IEC, and 1.0 −IEC FWs, respectively. A graphical illustration of the FWHM results can be seen in Fig. 5.

**Table 1:** Field width FWHM comparison (mm). Comparison between the gold standard reference values (GS Ref.) and measured FWHMs for the nominal field width (FW) dose profiles in the dynamic jaw motion beam model. All values are shown in mm unless specified otherwise

Nominal FW	Symmetric FWs				Asymmetric FWs		
	5.0	2.5	2.0	1.0	2.5 ± IEC	2.0 ± IEC	1.0 ± IEC
GS Ref.	51.44	25.55	18.51	10.79	25.44	18.19	10.36
Measured (mean ± std)	51.51 ± 0.05	25.64 ± 0.04	18.59 ± 0.04	10.83 ± 0.06	25.52 ± 0.06 (+) 25.51 ± 0.05 (−)	18.29 ± 0.08 (+) 18.26 ± 0.07 (−)	10.43 ± 0.10 (+) 10.40 ± 0.05 (−)
Difference	0.08 (0.14%)	0.09 (0.35%)	0.08 (0.44%)	0.04 (0.33%)	0.09 (0.34%) (+) 0.07 (0.28%) (−)	0.10 (0.55%) (+) 0.07 (0.39%) (−)	0.07 (0.67%) (+) 0.04 (0.39%) (−)

The beam profile constancy results are reported in Table 2. Beam profile constancy evaluated along the FW profile peaks was observed to be very high with 99.8% (*n* = 838) of individual measurements within 1% of baseline. The beam profile constancy evaluated across the full FW profile resulted in notable differences between symmetric and asymmetric FWs. For the symmetric FWs, the full profile results were within 3% of baseline in 95.2% (*n* = 320) of measurements, while only 63.9% (*n* = 322) of asymmetric FW measurements were within the 3% criteria. Despite this, 94.8% (*n* = 398) of asymmetric FW measurements, excluding the 1.0 −IEC FW, were within an expanded criteria of 4%. The full beam profile constancy for the 1.0 −IEC FW was greater than 5% of baseline in 20.2% (*n* = 17) of measurements.

**Table 2:** Field width constancy (%). Results for the beam profile constancy (mean ± std) of the profile measurements with the gold standard reference FW profiles as the baseline. Full profile constancy was evaluated between the 5% profile values and peak constancy was evaluated between the 95% values

Nominal FW	Symmetric FWs				Asymmetric FWs		
	5.0	2.5	2.0	1.0	2.5 ± IEC	2.0 ± IEC	1.0 ± IEC
Full profile constancy	0.56 ± 0.20	0.84 ± 0.21	1.02 ± 0.28	1.59 ± 0.52	1.12 ± 0.31 (+) 0.97 ± 0.32 (−)	1.82 ± 0.57 (+) 1.49 ± 0.50 (−)	1.97 ± 0.57 (+) 2.43 ± 0.86 (−)
Peak constancy	0.14 ± 0.02	0.12 ± 0.04	0.15 ± 0.05	0.24 ± 0.11	0.22 ± 0.13 (+) 0.12 ± 0.07 (−)	0.28 ± 0.21 (+) 0.10 ± 0.06 (−)	0.20 ± 0.10 (+) 0.12 ± 0.05 (−)

## 4 DISCUSSION

The use of a common beam model for dose calculation in TomoTherapy means that individual machine beam parameters invariably have slight deviations from the model. The average systematic difference of 0.07 mm observed in this study (deducible from Table 1) was consistent with the original FWHM results at the time of TomoEDGE™ commissioning, that is, 0.06 mm, which was permissible within the current manufacturer guidelines for FW acceptance. Furthermore, such a systematic difference in FWHM does not compromise clinical treatments, which were verified with patient‐specific QA. The systematic difference was not attributable to the measurements being made in solid water. In fact, comparison of the study's average FWHM data against annual watertank measurements resulted in a median difference of 0.12% (−0.05 to 0.67%) for all field widths. However, some criticalities emerged, such as the noncompliance of the smaller asymmetric FWs that was often evident. Indeed, while the FWHM results indicate that the recommended 1% tolerance limit was well respected for the symmetric 5.0, 2.5, 2.0 FWs and asymmetric 2.5 ±IEC, 2.0 −IEC FWs; the 2.0 +IEC FW, and 1.0 FWs, symmetric and asymmetric, were unable to consistently satisfy the AAPM TG‐148 1% recommendation in one quarter of the measurements (Fig. 5). The reason for their frequent noncompliance is most likely due to a combination of the following three concurring aspects. First, as mentioned in the TG‐148 report, smaller fields are affected more greatly as a percentage of their FWHM for a given difference in absolute position. Smaller fields are therefore more susceptible to factors extraneous to the jaw movement, for example, noise in couch velocity during the couch translation. Second, the dynamic jaw encoders inherently have an absolute position tolerance of 50 μm (0.05 mm),^1^ which is already half of the stipulated 1% tolerance limit for the 1.0 cm FW. Last, commissioning and calibration of the dynamic jaws is performed using the manufacturer's procedure, which utilizes gamma analysis in comparison of measurement profiles with those of the beam model.^8^ The manufacturer's established acceptance criteria is a resultant *γ*‐index < 1 with a global dose difference (DD) of 3% and distance‐to‐agreement (DTA) of 0.5 mm for the asymmetric fields.^1^ This DTA criterion for asymmetric FWs was chosen by the manufacturer because the profiles share either an inferior or superior edge with the 5.0 cm jaw setting, which is calibrated to be within 1% of its nominal FW, that is, 0.5 mm. In consequence, the accuracy of the FWHM of the asymmetric profiles is dependent on the accuracy of the 5.0 cm jaw setting, which corresponds to 5% of the 1.0 cm opening. Furthermore, it is evident that the current AAPM recommended tolerance limit of 1% of FW is more stringent than the gamma analysis DTA criterion applied by the manufacturer in calibration of the TomoEDGE™ asymmetric FWs, justifying the frequent noncompliance of smaller asymmetric FWs.

Beam profile constancy is an important parameter that ensures a consistent beam profile. This parameter has usually been implemented concerning field flatness and symmetry for large fields; however, AAPM TG‐142 has also recommended its use for an increased sensitivity to beam shape changes. In contrast, due to high gradients and lack of flatness in small fields, the AAPM TG‐135 report on quality assurance of robotic radiosurgery recommends measuring at least three radial locations within the central portion of the beam as a constancy check.^9^ The purpose of including the AAPM TG‐142 definition of beam profile constancy in this study, even though TomoTherapy FWs are small, was to increase sensitivity to possible changes in beam shape while further enabling assessment over a larger area than a few radial locations. In addition, radial measurements are not sensitive to possible shifts in the asymmetric beam profile shape. The results for the peak region demonstrate the upper portion of the profiles is very consistent to the model, which could indicate a relative insensitivity to the observed uncertainties in jaw position. Beam profile constancy results for the full profiles showed that the profile shapes were locally consistent to the model to within 3–4% for all FWs with exception to the 1.0 −IEC FW (Table 2). Interestingly, the beam profile constancy for the 1.0 −IEC FW demonstrates that the beam shape was noticeably different from that of the model although FWHM comparisons were within the AAPM tolerance in 92.9% of its measurements. This discrepancy indicates a slight skew in the longitudinal direction of the asymmetric beam profile. Specifically, the observed skew measured 0.08 mm at the 10% value and −0.07 mm at the 90% value, which resulted in a visible tilt of the profile when compared to its reference. The tilt in the longitudinal beam profile is likely caused by the relative position of the radiation source in relation to the jaw position for the −1.0 IEC FW. Presently, the asymmetric jaw position‐to‐source alignment is not a parameter checked within machine QA and therefore, no tolerance limit currently exists defining its agreement with reference position.

## 5 CONCLUSION

This 2‐yr study has shown that the measured FW dose profiles of the new beam model respect the current AAPM TG‐148 FWHM tolerance limit of 1% in most cases. However, it is important to recognize that although TomoEDGE™ utilizes a different beam model with more FW profiles, TomoEDGE™ must be evaluated against the current standards for QA of the TomoTherapy system. Thus, it is possible that the introduction of the asymmetric FWs requires dosimetric evaluation similar to that which was performed in the Balog et al. study from which the original 1% FWHM tolerance was established. Furthermore, the establishment of an appropriate QA tolerance for the asymmetric FWs becomes more relevant with the recent development of motion management utilizing the dynamic jaws.^10^ The AAPM TG‐148 report for quality assurance of TomoTherapy was published prior to the introduction of dynamic jaw movement and the study suggests that the recommended tolerance limits could be revisited in lieu of the present technology. The study results have also shown that full beam profile constancy may be a useful parameter for detecting changes in the beam profile shape not detectable with FWHM comparisons.

In conclusion, this study has strictly attempted to assess the TomoEDGE™ technology in its accuracy and precision of jaw positioning. The next step should be to evaluate the clinical significance of the observed measurement deviations and reported discrepancies from current standard QA tolerance limits.

## CONFLICT OF INTEREST

The authors declare no conflict of interest.
